# Near-Space TOPSAR Large-Scene Full-Aperture Imaging Scheme Based on Two-Step Processing

**DOI:** 10.3390/s16081177

**Published:** 2016-07-27

**Authors:** Qianghui Zhang, Junjie Wu, Wenchao Li, Yulin Huang, Jianyu Yang, Haiguang Yang

**Affiliations:** School of Electronic Engineering, University of Electronic Science and Technology of China, Chengdu 611731, China; qh_zhang09@163.com (Q.Z.); lwc6@163.com (W.L.); yulinhuang@uestc.edu.cn (Y.H.); jyyang@uestc.edu.cn (J.Y.); yanghaiguang@uestc.edu.cn (H.Y.)

**Keywords:** SAR, near-space SAR, TOPS, sustained large-scene imaging, two-step processing, modified two-step processing

## Abstract

Free of the constraints of orbit mechanisms, weather conditions and minimum antenna area, synthetic aperture radar (SAR) equipped on near-space platform is more suitable for sustained large-scene imaging compared with the spaceborne and airborne counterparts. Terrain observation by progressive scans (TOPS), which is a novel wide-swath imaging mode and allows the beam of SAR to scan along the azimuth, can reduce the time of echo acquisition for large scene. Thus, near-space TOPS-mode SAR (NS-TOPSAR) provides a new opportunity for sustained large-scene imaging. An efficient full-aperture imaging scheme for NS-TOPSAR is proposed in this paper. In this scheme, firstly, two-step processing (TSP) is adopted to eliminate the Doppler aliasing of the echo. Then, the data is focused in two-dimensional frequency domain (FD) based on Stolt interpolation. Finally, a modified TSP (MTSP) is performed to remove the azimuth aliasing. Simulations are presented to demonstrate the validity of the proposed imaging scheme for near-space large-scene imaging application.

## 1. Introduction

Near space is defined as the atmospheric region from about 20 km to 100 km above the Earth’s sea level, which is usually not accessible to satellites or conventional maneuvering aircrafts [1]. Synthetic aperture radar (SAR) equipped on near-space platform, which is free of the constraints of the orbit mechanism and weather conditions, has drawn widespread attention for its unique capabilities such as short revisiting cycle compared with spaceborne SAR, and sustained large-scene imaging compared with airborne SAR [2,3,4,5,6,7,8].

Persistent imaging for large scene is pressing for situations such as environmental monitoring and public security [9,10]. Due to the minimum antenna constraint, the ratio of swath width to azimuth resolution has a constraint as follows [2]:

(1) $$\begin{array}{c} \begin{array}{c} \frac{W_{s}}{\rho_{a}}\leq \frac{c}{2\cdot v_{s}\cdot \sin\left(\theta \right)} \end{array} \end{array}$$

where $W_{s}$ is swath width, $\rho_{a}$ is azimuth resolution, *c* is speed of light, $v_{s}$ is platform velocity, and *θ* is incidence angle. Generally, $c/v_{s}$ is nearly constant at 20,000 for low-Earth-orbit (LEO) satellites and typically in the range of 300,000–750,000 for airplanes. As near-space vehicles can fly at a speed ranging from almost stationary to 100 m/s, the $c/v_{s}$ will be greater than 3,000,000, which means the SAR carried by near-space platform is almost free of the constraint of the minimum antenna area. Thus, the near-space SAR can provide a more flexible opportunity for sustained high-resolution wide-swath imaging compared with spaceborne and airborne counterparts [2].

**Figure 1:**
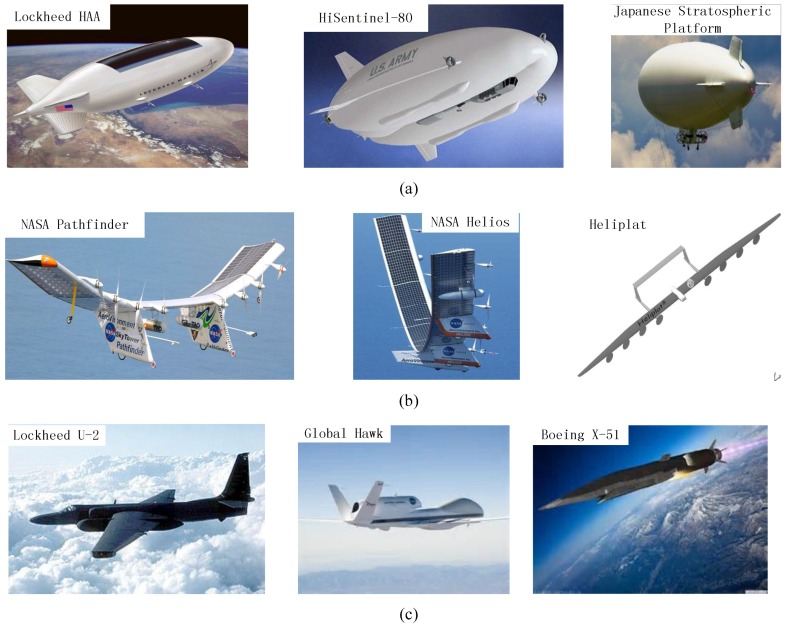
Typical near-space platforms. (**a**) near-space airship (NSA); (**b**) near-space long-endurance low-speed-maneuvering vehicle (NLLV); (**c**) near-space short-endurance high-speed-maneuvering vehicle (NSHV).

Although near-space SAR is a promising candidate for future microwave remote sensing missions, two key problems should be considered and addressed before the near-space SAR comes true. The first one is the near-space platform techniques, the other is the operation mode design and the corresponding imaging scheme. For the first problem, as the potential value of the near space becomes more and more clear, many studies related to the near-space platform have been reported [11,12,13,14,15,16,17]. Near-space platforms can be clarified into three types: near-space airship (NSA), near-space long-endurance low-speed-maneuvering vehicle (NLLV) and near-space short-endurance high-speed-maneuvering vehicle (NSHV), as shown in Figure 1. Benefitting from the lighter-than-air gas inside its huge body and the solar panels on its surfaces, NSA can potentially float in the air up to several years without landing on the ground for supplements or maintenance. However, even for the NSA equipped with electrical engines and steering propellers, it is difficult to drift on wind with stably or quasi-stably velocity. Thus, NSA is more suitable for the applications like communication/broadcast relay and stationary radar surveillance/detection, but not for SAR. The third type of near-space platform, NSHV, is quite suitable for quick-response surveillance for the high speed bringing by its powerful jet engine and perfect streamlined body. However, its endurance is much shorter than that of NSA due to the high fuel-consumption of high-speed maneuvering. Only the second type of near-space platform, NLLV, is the most suitable and promising candidate to carry the future near-space SAR for sustained large-scene microwave imaging mission. The reasons are that the NLLV possesses two important properties simultaneously, i.e., the relatively long endurance (ranging from several weeks to several years) bringing by its high-efficient solar panels, and the stable maneuvering speed (up to tens of meters per second) bringing by its advanced electrical engines and well designed streamlined body.

As for the operation mode and the corresponding imaging scheme, terrain observation by progressive scans (TOPS), which is a novel wide-swath imaging mode and allows the beam of SAR to scan along azimuth direction, can reduce the time of echo acquisition for large scene [18]. Thus, near-space TOPS-mode SAR (NS-TOPSAR) brings a new opportunity for sustained and efficient large-scene imaging.

Unfortunately, the azimuth beam scanning of NS-TOPSAR leads to the variation of the Doppler centroid of targets with the same range [19]. It causes the total Doppler bandwidth of the echo signal to be much wider than that of a single target, resulting in serious aliasing in the Doppler domain of the echo. A possible solution for the problem of aliasing is using a high pulse repetition frequency (PRF) during the data acquisition. However, a high PRF may cause not only the range ambiguity, but also the increasing of the data storage and downlink load. Thus, the raw data of a practical TOPSAR is usually aliased in Doppler domain.

To process the TOPS raw data with Doppler aliasing, Prats et al. [20] proposed a method based on sub-aperture formation. However, the amount of sub-apertures (i.e., the number of the blocks into which the raw data are divided in range and/or azimuth time domain) will be great in the case of low PRF, leading to the unnecessary increasing of the data volume. Hence, developing more efficient full-aperture imaging schemes is significant for the processing of the NS-TOPSAR data. Francesco et al. [18] proposed an ωkA imaging scheme with mosaic operation in azimuth. In this scheme, the “mosaiking” of the Doppler spectrum is introduced to transform the aliasing from the Doppler domain to the azimuth time domain (TD), then the conventional ωkA is adopted to focus the targets, finally a modified mosaiking operation is conducted to resolve the azimuth folding (i.e., the aliasing of the image in azimuth TD) problem. The main drawback of the mosaic method is that it needs to duplicate and down-sample the Doppler spectrum, which is not only inefficient but also leads to the degradation of the quality of the impulse response (IPR). Engen et al. [21] proposed another ωkA with moving band chirp-Z transform (MBCZT) and inverse MBCZT to resolve the Doppler aliasing and azimuth folding respectively. Guang-Cai Sun et al. [22] proposed a generalized polar format algorithm (PFA) imaging scheme, in which the deramping operation is adopted to reduce the total Doppler bandwidth of the raw data, the generalized PFA (GPFA) is proposed to focus the targets and a scaling operation is adopted to reduce the time-span of the resulted image to avoid the azimuth folding. However, the GPFA needs two-dimensional (2D) interpolation, which is not efficient [23] compared to the ωkA which only needs 1D interpolation.

Besides, Guang-Cai Sun et al. [24] proposed a chirp scaling algorithm (CSA) based on two-step processing (TSP, originally proposed in [25] to process Spotlight SAR data) and SPECtral ANalysis (SPECAN) [26]. In this scheme, TSP is adopted to transform the aliasing from the Doppler domain to the azimuth TD, then the CSA (without azimuth compression) is conducted to perform range compression, second range compression (SRC) and range cell migration correction (RCMC). Finally, an azimuth scaling operation is adopted to transform the hyperbolic phase curve to a quadratic phase one, followed by SPECAN operation to perform azimuth compression. Wei Xu et al. [27] concluded the available preprocessing methods of Doppler aliasing elimination for TOPSAR and proposed four imaging schemes: CSA with mosaic postprocessing, extended CSA (ECSA) with SPECAN, modified ECSA based on chirp-z transform (CZT) and baseband azimuth scaling (BAS) algorithm. They share the same preprocessing method (i.e., TSP) to eliminate the Doppler aliasing of the raw data and CSA to perform range compression, SRC and RCMC. The differences among the four imaging schemes lie in the successive steps after the CSA operation. For instance, in the first imaging scheme, a mosaiking operation similar to that in [18] is adopted to resolve the azimuth folding after azimuth compression. While the second imaging scheme is the same as that in [24], except for the miner difference in the way of choosing the reference distance of the scaling factor. The third imaging scheme is similar to the second one, except that the scaling operation is realized by more efficient scaled Fourier transform (SCFT). The last imaging scheme (i.e., BAS algorithm), also published earlier in [28] with the same authors, utilizes the BAS operation similar to that in [20] to remove the azimuth folding, except that a slant-range-dependent azimuth scaling factor instead of a constant one is chosen.

In this paper, a full-aperture ωkA for NS-TOPSAR based on TSP is proposed. In this scheme, TSP is adopted to eliminate the Doppler aliasing of the echo firstly by transforming the aliasing from Doppler domain to azimuth TD. Then, the data is focused on the 2D frequency domain (FD) based on Stolt interpolation [29]. Finally, a modified TSP (MTSP) is proposed to resolve the azimuth folding by transforming the aliasing from the azimuth TD back to the azimuth FD. Similar to the original TSP, the MTSP needs only one fast Fourier transform (FFT), one inverse FFT (IFFT) and three phase multiplications, and, thus, is very efficient.

The rest of the paper is organized as follows. In Section 2, the data acquisition geometry model and echo signal mathematical model of NS-TOPSAR are given, then the main property of the echo signal is analyzed. In Section 3, the imaging scheme is presented and analyzed in detail. In Section 4, simulations are presented. Finally, Section 5 draws conclusions.

## 2. NS-TOPSAR

### 2.1. Geometry Model and Echo Signal Model

**Figure 2:**
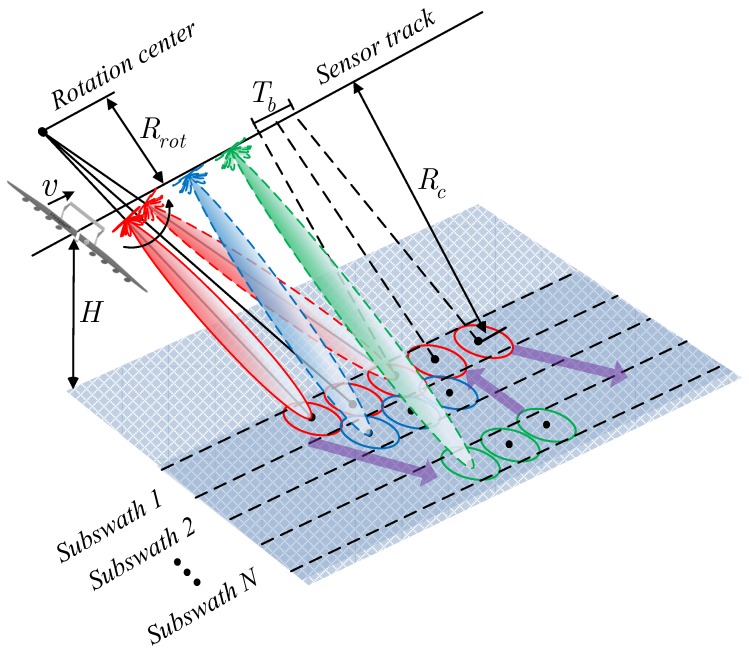
Geometry model of near-space terrain observation by progressive scans SAR (NS-TOPSAR).

As shown in Figure 2, NS-TOPSAR moves straight with constant velocity *v* and height *H*. The total swath is divided into several subswaths. During the data acquisition, the beam of NS-TOPSAR is steered from aft to fore along azimuth within a subswath, which is called a *burst*. After a burst is finished, the beam is steered to scan the next subswath. The process is repeated until the burst of *Subswath N* is finished. Then, the beam is steered back to scan *Subswath 1*. The image of a large scene is obtained after succussive data processing, which is shown in the following section.

Assuming that linear frequency modulation (LFM) pulse is transmitted, the associated echo signal of a point target of NS-TOPSAR can be expressed as follows:

(2) $$\begin{array}{cc} & S_{0}\left(\tau ,\eta \right)\\ = & rect\left(\frac{\eta }{T_{b}}\right)w_{r}\left(\frac{\tau -\frac{2R\left(\eta \right)}{c}}{T_{r}}\right)w_{a}\left(\frac{\eta -\eta_{c}}{T_{a}}\right)\exp\left\{j\pi K_{r}{\left[\tau -\frac{2R\left(\eta \right)}{c}\right]}^{2}\right\}\exp\left[-j\frac{4\pi }{\lambda }R\left(\eta \right)\right] \end{array}$$

where

$$\begin{array}{c} rect(\cdot ):\;\text{rectangular}\;\text{window}\;\text{function},\\ w_{r}\left(\cdot \right),w_{a}\left(\cdot \right):\;\text{range}\;\text{and}\;\text{azimuth}\;\text{window,}\;\text{respectively},\\ \tau ,\eta :\;\text{fast}\;\text{time}\;\text{and}\;\text{slow}\;\text{time,}\;\text{respectively},\\ \eta_{c}:\;\text{azimuth}\;\text{center}\;\text{time}\;\text{of}\;\text{the}\;\text{target},\\ T_{r}:\;\text{transmitted}\;\text{pulse}\;\text{duration},\\ T_{a}:\;\text{synthetic}\;\text{aperture}\;\text{time},\\ T_{b}:\;\text{burst}\;\text{duration},\\ c:\;\text{speed}\;\text{of}\;\text{light},\\ \lambda :\;\text{wavelength}\;\text{of}\;\text{the}\;\text{carrier},\\ K_{r}:\;\text{modulation}\;\text{rate}\;\text{of}\;\text{LFM}\;\text{pulse},\\ R\left(\eta \right):\;\text{range}\;\text{history}. \end{array}$$

### 2.2. Echo Signal Property

The azimuth time-frequency property is studied here because it is critical for the design of the imaging scheme for NS-TOPSAR.

The Doppler frequency of a target of NS-TOPSAR can be obtained as follows:

(4) $$\begin{array}{c} \begin{array}{c} f_{d}\left(\eta \right)=-\frac{2}{\lambda }\frac{\partial R\left(\eta \right)}{\partial \eta }\approx -\frac{2v^{2}\left(\eta -\eta_{0}\right)}{\lambda R_{p}} \end{array} \end{array}$$

where $\eta_{0}$ denotes the zero-Doppler time and $R_{p}$ presents the nearest slant range of the target.

**Figure 3:**
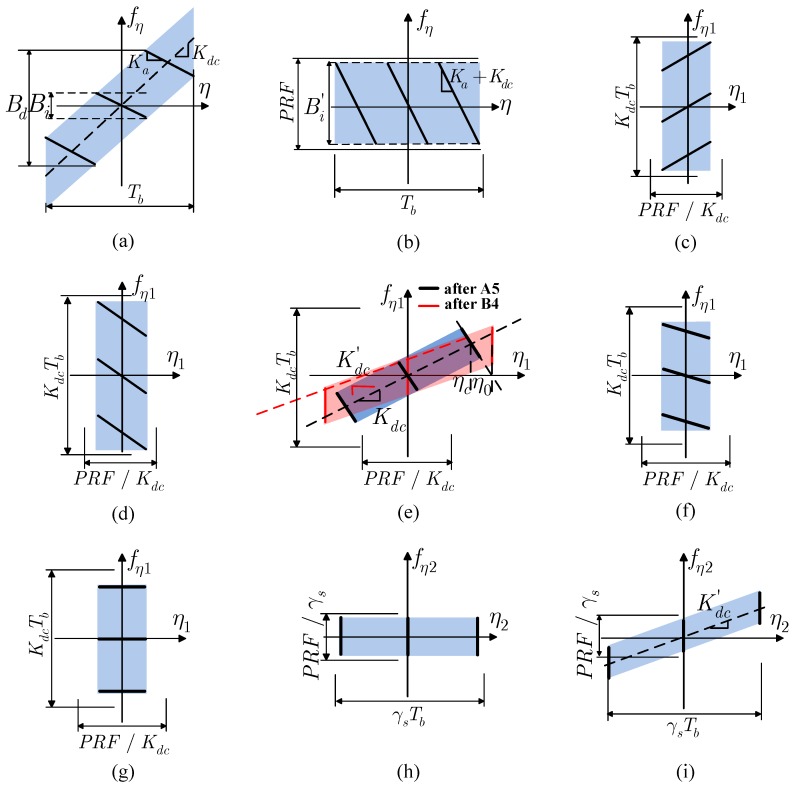
Azimuth time frequency diagram (TFD). (**a**) echo signal; (**b**) after A1; (**c**) after A2; (**d**) after A3; (**e**) after A5 and after B4; (**f**) after C1; (**g**) after C3; (**h**) after C4; (**i**) after C5.

From Equation (3), the time-frequency diagram (TFD) of the azimuth echo signal can be schematically drawn out, which are shown in Figure 3a (only three targets are shown, one in the center and two in the boundary of a burst scene), where

$$\begin{array}{c} K_{a}:\;\text{Doppler}\;\text{modulation}\;\text{rate,}\\ K_{dc}:\;\text{the}\;\text{Doppler}\;\text{centroid}\;\text{change}\;\text{rate,}\\ \;\text{i.e.,}\;\text{the}\;\text{gradient}\;\text{of}\;\text{the}\;\text{Doppler}\;\text{centroid}\;\text{with}\;\text{respect}\;\text{to}\;\eta ,\\ B_{i}:\;\text{instant}\;\text{Doppler}\;\text{bandwidth,}\\ \;\text{i.e.,}\;\text{the}\;\text{Doppler}\;\text{bandwidth}\;\text{of}\;a\;\text{single}\;\text{target},\\ B_{d}:\;\text{total}\;\text{Doppler}\;\text{bandwidth,}\\ \;\text{i.e.,}\;\text{the}\;\text{total}\;\text{Doppler}\;\text{bandwidth}\;\text{of}\;a\;\text{burst},\\ R_{c}:\;\text{the}\;\text{nearest}\;\text{slant}\;\text{range}\;\text{of}\;\text{the}\;\text{burst}\;\text{scene}\;\text{center},\\ R_{rot}:\;\text{the}\;\text{nearest}\;\text{slant}\;\text{range}\;\text{of}\;\text{the}\;\text{rotation}\;\text{center}. \end{array}$$

From Figure 3a, it can be concluded that the scanning of the beam of NS-TOPSAR along the azimuth direction results in a curious situation that the total Doppler bandwidth of a burst is much wider than the instant Doppler bandwidth. However, the PRF of NS-TOPSAR is usually set to be several times greater than $B_{i}$ and much less than $B_{d}$. Thus, the echo signal is usually seriously aliased in the Doppler domain.

## 3. Imaging Scheme

**Figure 4:**
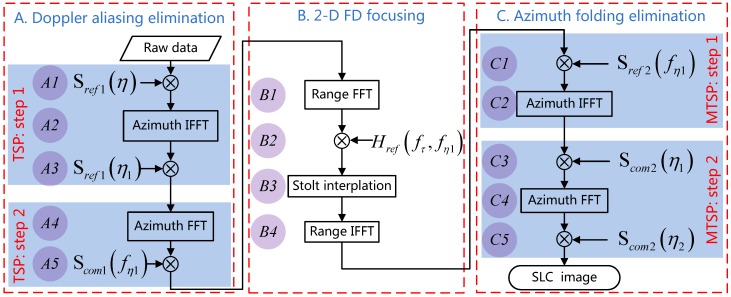
Flowchart of proposed imaging scheme.

According to the property of the echo signal discussed in Section 2.2, we propose a full-aperture imaging scheme based on TSP for NS-TOPSAR large-scene imaging. The flowchart of the proposed imaging scheme for burst data processing is shown in Figure 4. The scheme is divided into three parts marked “A”, “B” and “C” from left to right in Figure 4, respectively, which are discussed in detail as follows.

### 3.1. Doppler Aliasing Elimination by TSP

As discussed in Section 2.2, the Doppler centroid of the targets with the same range varies approximately linearly with respect to the azimuth time, which implies that the SPECAN technique can be adopted to deramp the Doppler centroid with the following phase function:

(4) $$\begin{array}{c} \begin{array}{c} S_{ref1}\left(\eta \right)=\exp\left(-j\pi K_{dc}\eta_{}^{2}\right) \end{array} \end{array}$$

Thus, TSP based on the SPECAN technique is adopted here to eliminate the Doppler aliasing of the echo signal. It consists of the following steps:

STEP 1 of TSP: convolve the echo signal with the reference phase function shown in Equation (4); then, we obtain:

(5) $$\begin{array}{c} \begin{array}{cc} S_{1}\left(\tau ,\eta_{1}\right)= & \exp\left(-j\pi K_{dc}\eta_{1}^{2}\right)\times \int S_{0}\left(\tau ,\eta \right)\exp\left(-j\pi K_{dc}\eta_{}^{2}\right)\exp\left(j2\pi K_{dc}\eta_{1}\eta \right)d\eta \end{array} \end{array}$$

where $\eta_{1}$ denotes the new azimuth time variable after the convolution. According to SPECAN, the convolution in Equation (5) can be efficiently computed by phase multiplications and inverse fast Fourier transform (IFFT) if the following relation is set to be valid:

(6) $$\begin{array}{c} \begin{array}{c} K_{dc}\eta_{1}=f_{\eta } \end{array} \end{array}$$

According to the Fourier transform theory, the azimuth spectrum of $S_{1}\left(\tau ,\eta_{1}\right)$ in Equation (5) can be expressed as follows:

(7) $$\begin{array}{cc} S_{1}\left(\tau ,f_{\eta 1}\right)= & FT_{az}\left\{S_{0}\left(\tau ,\eta_{1}\right)\right\}\cdot FT_{az}\left\{S_{ref1}\left(\eta_{1}\right)\right\}\\ = & S_{0}\left(\tau ,f_{\eta 1}\right)\cdot \exp\left(j\pi f_{\eta 1}^{2}/K_{dc}\right) \end{array}$$

where $f_{\eta 1}$ is the frequency variable corresponding to $\eta_{1}$, and $FT_{az}\left\{\cdot \right\}$ denotes the Fourier transform along the azimuth direction.

The Doppler spectrum of the echo signal can be obtained by compensating the exponential term on the right side of Equation (7). Thus, the next step of TSP is derived as follows.

STEP 2 of TSP: transform the result of STEP 1 to the range-Doppler domain by azimuth FFT and then multiply the result by the following phase function:

(8) $$\begin{array}{c} \begin{array}{c} S_{com1}\left(f_{\eta 1}\right)=\exp\left(-j\pi f_{\eta 1}^{2}/K_{dc}\right) \end{array} \end{array}$$

As shown in Figure 4, A1–A3 belong to the STEP 1 of TSP, and A4–A5 belong to STEP 2. The azimuth TFD of the signal during the processing is shown in Figure 3a–e, from which it can be concluded that the new equivalent azimuth sampling frequency increases from the original PRF to $K_{dc}T_{b}$ (≈$B_{d}$). In other words, the Doppler aliasing is eliminated.

### 3.2. 2D FD Focusing by ωkA

After the processing of Part A (cf. the left part of Figure 4), the Doppler aliasing of the echo signal is eliminated; thus, the 2D FD focusing technique ωkA can be adopted to focus the targets. Firstly, the signal produced by Part A is transformed to 2-D FD, and then a bulk compression is conducted by multiplying the following reference phase function:

(9) $$\begin{array}{c} \begin{array}{c} H_{ref}\left(f_{\tau },f_{\eta 1}\right)=\exp\left(j\pi \frac{f_{\tau }^{2}}{K_{r}}\right)\exp\left(j\frac{4\pi R_{c}}{c}\sqrt{{\left(f_{\tau }+f_{0}\right)}^{2}-\frac{c^{2}f_{\eta 1}^{2}}{4v^{2}}}\right) \end{array} \end{array}$$

where $f_{\tau }$ is the frequency variable corresponding to *τ*.

The bulk compression focuses the target located in the center of a burst scene perfectly, while the targets deviating from the scene center are not fully focused due to the space variance. Therefore, a Stolt interpolation is then conducted to remove the residual phase modulation of those targets with the following mapping relationship:

(10) $$\begin{array}{c} \begin{array}{c} \sqrt{{\left(f_{\tau }+f_{0}\right)}^{2}-\frac{cf_{\eta 1}^{2}}{4v^{2}}}=f_{\tau 1} \end{array} \end{array}$$

where $f_{\tau 1}$ denotes the new range frequency after Stolt interpolation:

### 3.3. Azimuth Folding Elimination by MTSP

After the processing of Part B (cf. the middle part of Figure 4), all targets have been fully focused. However, azimuth folding will occur if the signal is simply transformed to 2D TD by IFFT. The reasons are presented as follows. As shown in Figure 3a–e, during Part A (cf. the left part of Figure 4), the Doppler aliasing of the echo signal is eliminated, while the azimuth time span is folded from $T_{b}$ to $PRF/K_{dc}$. In addition, from Figure 3e, it can be seen that after the processing of Part B, the targets are located in their zero-Doppler position, which results in the azimuth time span of the targets expanding from $T_{b}$ to $\gamma_{s}T_{b}$, where,

(11) $$\begin{array}{c} \begin{array}{c} \gamma_{s}=\frac{R_{rot}+R_{c}}{R_{rot}} \end{array} \end{array}$$

denotes the TOPS coefficient. These factors result in the azimuth folding of the focused signal produced by Part B.

Actually, the azimuth folding phenomenon can be seen as aliasing of the signal in azimuth TD. Inspired by the above-mentioned TSP, which is adopted to eliminate the Doppler aliasing of the raw data of NS-TOPSAR, we propose a modified TSP (MTSP) to eliminate the azimuth folding of the resulted signal of Part B (cf. the middle part of Figure 4). Different from the original TSP, the first step of MTSP involves reference phase function multiplication in the range-Doppler domain to introduce a pre-distortion phase to the signal and azimuth IFFT to transform the signal to 2D TD. The second step is in convolution with the compensation function. The detail of MTSP is illustrated as follows.

Step 1 of MTSP: multiple the resulted signal of Part B by the following reference phase function:

(12) $$\begin{array}{c} \begin{array}{cc} S_{ref2}\left(f_{\eta 1}\right) & =\exp\left(j\pi f_{\eta 1}^{2}/K_{dc}^{{}^{\prime }}\right) \end{array} \end{array}$$

where,

(13) $$\begin{array}{c} \begin{array}{c} K_{dc}^{{}^{\prime }}=\frac{K_{dc}}{\gamma_{s}} \end{array} \end{array}$$

denotes the Doppler centroid change rate of the signal produced by Part B. Then, the signal is transformed into 2D TD by azimuth IFFT.

Step 2 of MTSP: in order to compensate the phase function multiplication in step 1 of MTSP above, we convolve the resulted signal of the step 1 of MTSP with the following phase function:

(14) $$\begin{array}{c} \begin{array}{cc} S_{com2}\left(\eta_{1}\right) & =\exp\left(j\pi K_{dc}^{{}^{\prime }}\eta_{1}^{2}\right) \end{array} \end{array}$$

Then, we obtain:

(15) $$\begin{array}{cc} S_{out}\left(\tau_{1},\eta_{2}\right) & =\int S_{2}\left(\tau_{1},\eta_{1}\right)\exp\left(j\pi K_{dc}^{{}^{\prime }}{\left(\eta_{2}-\eta_{1}\right)}^{2}\right)d\eta_{1}\\ & =\exp\left(j\pi K_{dc}^{{}^{\prime }}\eta_{2}^{2}\right)\int S_{2}\left(\tau_{1},\eta_{1}\right)\exp\left(j\pi K_{dc}^{{}^{\prime }}\eta_{1}^{2}\right)\exp\left(-j2\pi K_{dc}^{{}^{\prime }}\eta_{2}\eta_{1}\right)d\eta_{1}\\ & =S_{com2}\left(\eta_{2}\right)\int S_{2}\left(\tau_{1},\eta_{1}\right)S_{com2}\left(\eta_{1}\right)\exp\left(-j2\pi K_{dc}^{{}^{\prime }}\eta_{2}\eta_{1}\right)d\eta_{1} \end{array}$$

where $S_{2}\left(\tau_{1},\eta_{1}\right)$ denotes the resulted signal of the step 1 of MTSP (here $\tau_{1}$ denotes the range time variable corresponding to $f_{\tau 1}$), and $S_{out}\left(\tau_{1},\eta_{2}\right)$ denotes the output signal of the convolution, and

(16) $$\begin{array}{c} \begin{array}{cc} S_{com2}\left(\eta_{2}\right) & =\exp\left(j\pi K_{dc}^{{}^{\prime }}\eta_{2}^{2}\right) \end{array} \end{array}$$

Let the new azimuth time variable after the convolution, $\eta_{2}$, satisfy the following relationship:

(17) $$\begin{array}{c} \begin{array}{c} \eta_{2}=\frac{f_{\eta 1}}{K_{dc}^{{}^{\prime }}} \end{array} \end{array}$$

Then, Equation (18) becomes:

(18) $$\begin{array}{cc} S_{out}\left(\tau_{1},\eta_{2}\right) & =S_{com2}\left(\eta_{2}\right)\int S_{2}\left(\tau_{1},\eta_{1}\right)S_{com2}\left(\eta_{1}\right)\exp\left(-j2\pi f_{\eta_{1}}\eta_{1}\right)d\eta_{1}\\ & =S_{com2}\left(\eta_{2}\right)\cdot FT_{az}\left\{S_{2}\left(\tau_{1},\eta_{1}\right)\cdot S_{com2}\left(\eta_{1}\right)\right\} \end{array}$$

From Equation (21), it can be concluded that the convolution in Equation (18) can be realized efficiently by one phase multiplication, one azimuth FFT and one phase multiplication again, shown as “C3”, “C4” and “C5” in Figure 4, respectively.

The azimuth TFD during the processing of Part C is shown in Figure 3e–i. From Figure 3i, it can be concluded that the azimuth time span of the output image is expanded from $PRF/K_{dc}$ to $\gamma_{s}T_{b}$, which means that the azimuth folding is removed successfully.

## 4. Simulation

Simulation is provided here to verify the validity of the proposed imaging scheme. Furthermore, a brief performance comparison of the simulated NS-TOPSAR and typical spaceborne/airborne SAR systems with TOPS mode is drawn.

### 4.1. Simulation Parameters

**Figure 5:**
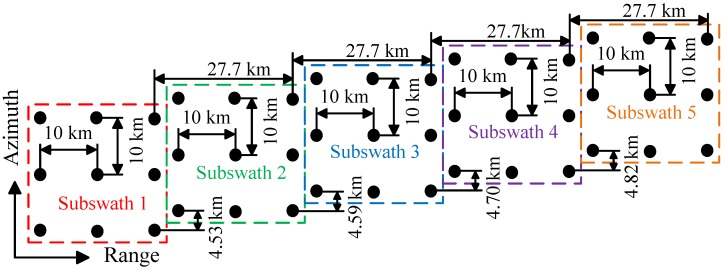
Diagram of simulated targets distribution.

Simulation parameters and the simulated point targets distribution are shown in Table 1 and Figure 5, respectively. In Table 1, the PRF which is adaptive to different subswaths, is merely about one-third of the total Doppler bandwidth. In Figure 5, the relative position of the targets within a subswath and the range shift between two adjacent subswaths are constant. While the azimuth shift between two adjacent subswaths is varying due to the difference in the PRF of each subswath.

### 4.2. Simulation Results

**Figure 6:**
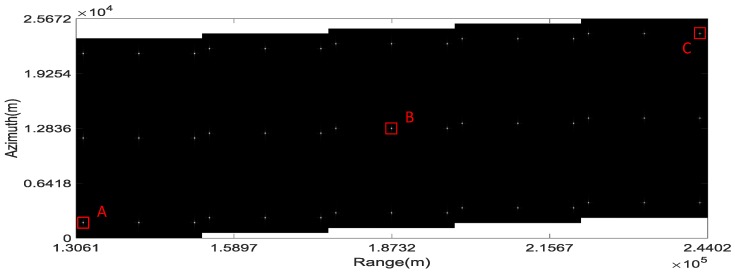
Imaging result of the proposed scheme.

The imaging result of the proposed scheme is shown in Figure 6. It can be concluded from Figure 6 that the simulated targets are focused well without aliasing and the total swath width is more than 100 km.

**Figure 7:**
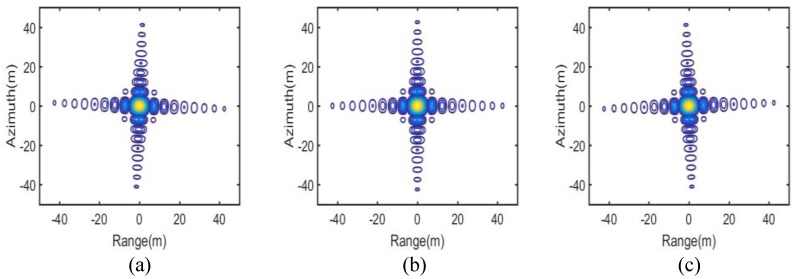
ImPulse Response (IPR) of the targets of the proposed scheme. (**a**) Target A; (**b**) Target B; and (**c**) Target C.

**Figure 8:**
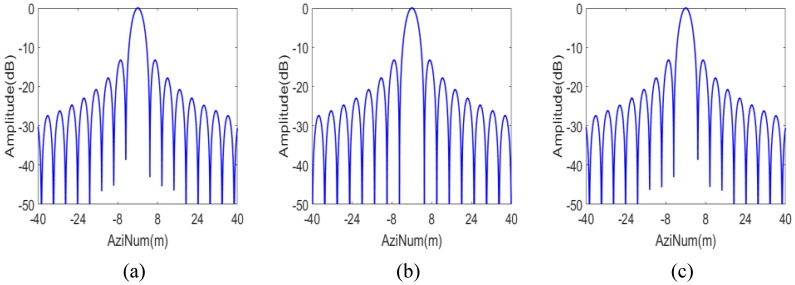
Azimuth profile of the targets of the proposed scheme. (**a**) Target A; (**b**) Target B; and (**c**) Target C.

The IPRs, azimuth profiles and the point target quality measurements of the three targets marked “A”, “B” and “C” in Figure 6 are shown in Figure 7 and Figure 8 and the upper part of Table 2, respectively. They provide numerical evidence for the validity of the proposed imaging scheme.

### 4.3. Imaging Scheme Comparison and Discussion

**Figure 9:**
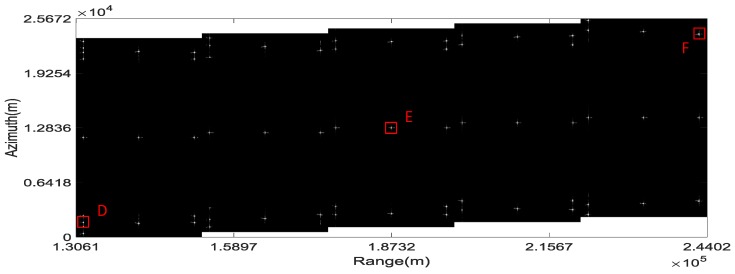
Imaging result of the scheme proposed in [18].

**Figure 10:**
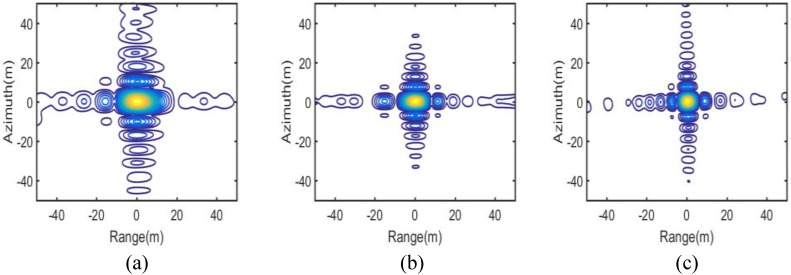
ImPulse Response (IPR) of the targets of the scheme proposed in [18]. (**a**) Target D; (**b**) Target E; and (**c**) Target F.

**Figure 11:**
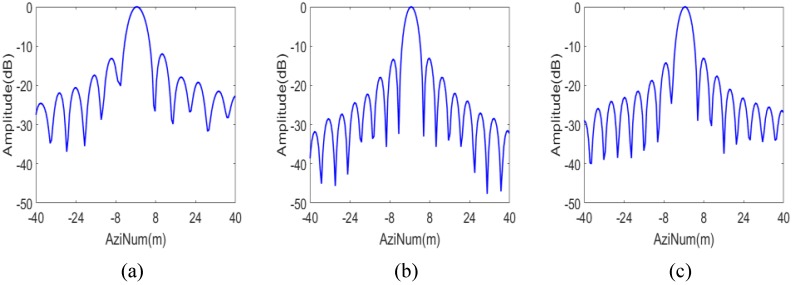
Azimuth profile of the targets of the scheme proposed in [18]. (**a**) Target D; (**b**) Target E; and (**c**) Target F.

As a contrast, the imaging result of the scheme proposed in [18] with the same simulation parameters is shown in Figure 9. It is clear in Figure 9 that ghost images of the nearest and farthest simulated targets of a burst appear due to the residual azimuth aliasing. The IPRs and azimuth profiles and the point target quality measurements for the targets marked “D”, “E” and “F” in Figure 9 are shown in Figure 10 and Figure 11 and the lower part of Table 2, respectively. From Figure 9 and Figure 11, it can be seen that the azimuth profile of target D is spoiled by nearby ghost images. While for target F, as the ghost image is relatively far away, the azimuth profile is much better, but still worse than that of target C.

According to Figure 6, Figure 7, Figure 8, Figure 9, Figure 10 and Figure 11 and Table 2, it can be concluded that the proposed imaging scheme have higher quality of IPR and less azimuth aliasing compared with that proposed in [18].

### 4.4. Performance Comparison of NS-TOPSAR and Spaceborne/Airborne TOPSAR

In order to assess the potential of the NS-TOPSAR more comprehensively, a brief performance comparison of the simulated NS-TOPSAR and typical spaceborne TOPSAR (Sentinel-1 [30,31,32]) and airborne TOPSAR (the TOPSAR in [24]) is drawn. The result is shown in Table 3, where the interferometric wide-swath (IW, standard operation mode) and the extra wide-swath (EW, another mode for extra wide-swath imaging) are two TOPS modes of Sentinel-1.

From Table 3, it can be concluded that the Spaceborne TOPSAR possesses the widest swathwidth, the coarsest resolution, the longest revisit time and the best endurance. While the airborne TOPSAR, which possesses the finest resolution, the narrowest swathwidth, the shortest revisit time and the worst endurance, goes to the other extreme. With more balanced performance in resolution, swathwidth, revisit time and endurance, future NS-TOPSAR can be a well supplement to the existing spaceborne and airborne counterparts, especially in areas requiring sustained large-scene imaging.

## 5. Conclusions

In this paper, the conception of near-space SAR is introduced. Then, the potential platforms and operation modes for this new kind of SAR are analyzed. An efficient full-aperture imaging scheme based on TSP for NS-TOPSAR large-scene imaging is proposed. TSP is adopted to remove the Doppler aliasing of the echo signal. Then, the resulting signal without Doppler aliasing is processed by the 2D FD focusing technique ωkA. Finally, the signal is processed by the proposed MTSP to remove the azimuth folding. Compared with existing imaging schemes, the azimuth folding elimination method proposed in this paper is not only very efficient because only FFT/IFFT and phase function multiplications are required, but also has storage economy because there is no need for zero-padding or data duplication. The validity and performance of the proposed scheme have been demonstrated by point target simulation. Furthermore, the characteristics of the proposed NS-TOPSAR are outlined by comparing their performance to that of spaceborne/airborne TOPSAR, which shows NS-TOPSAR is a promising and potential candidate for future sustained large-scene microwave imaging missions.

## Figures and Tables

**Table 1:** Simulation parameters.

General Parameters	Value				
Carrier frequency (GHz)	9				
Chirp bandwidth (MHz)	30				
Chirp duration (μs)	2				
Sample frequency (MHz)	36				
Antenna azimuth aperture (m)	1.7				
Platform velocity (m/s)	20				
Platform altitude (km)	25				
Number of subswath	5				
Total swath width (km)	115				
TOPS coefficient	5.2				
Swath-dependent parameters	Subswath				
	1	2	3	4	5
Slant range of center (km)	97	142	187	233	278
PRF (Hz)	113	60	41	32	27
Total Doppler bandwidth (Hz)	267	178	136	112	96

TOPS: terrain observation by progressive scan; PRF: pulse repetition frequency.

**Table 2:** Point target quality measurements.

Target	3 dB IRW (dB)	PSLR (dB)	ISLR (dB)
A	4.439	-13.262	-9.852
B	4.432	-13.266	-9.882
C	4.438	-13.264	-9.854
D	6.161	-12.558	-2.788
E	4.436	-13.092	-10.202
F	4.638	-13.164	-9.982

IRW: impulse response width; PSLR: peak side-lobe ratio; ISLR: integrated side-lobe ratio.

**Table 3:** Comparison of near-space, spaceborne and airborne TOPSAR.

	NS-TOPSAR	Sentinel-1		Airborne
		IW Mode	EW Mode	TOPSAR in [24]
Carrier frequency (GHz)	9	5.405	5.405	9.65
Altitude (km)	25	693	693	5
Resolution	4.5 × 4.5	5 × 20	20 × 40	2 × 1
(range × azimuth, m)				
Swathwidth (km)	100	250	400	20
Revisit time	several hours	6 days	6 days	within one hour
Endurance	up to a few years	tens of years	tens of years	up to a few days

TOPSAR: terrain observation by progressive scan (TOPS) SAR; IW: interferometric wideswath; EW: extreme wideswath.
